# On Simplified Global Nonlinear Function for Fitness Landscape: A Case Study of Inverse Protein Folding

**DOI:** 10.1371/journal.pone.0104403

**Published:** 2014-08-11

**Authors:** Yun Xu, Changyu Hu, Yang Dai, Jie Liang

**Affiliations:** Department of Bioengineering, University of Illinois at Chicago, Chicago, IL, United States of America; University of Michigan, United States of America

## Abstract

The construction of fitness landscape has broad implication in understanding molecular evolution, cellular epigenetic state, and protein structures. We studied the problem of constructing fitness landscape of inverse protein folding or protein design, with the aim to generate amino acid sequences that would fold into an *a priori* determined structural fold which would enable engineering novel or enhanced biochemistry. For this task, an effective fitness function should allow identification of correct sequences that would fold into the desired structure. In this study, we showed that nonlinear fitness function for protein design can be constructed using a rectangular kernel with a basis set of proteins and decoys chosen *a priori*. The full landscape for a large number of protein folds can be captured using only 480 native proteins and 3,200 non-protein decoys via a finite Newton method. A blind test of a simplified version of fitness function for sequence design was carried out to discriminate simultaneously 428 native sequences not homologous to any training proteins from 11 million challenging protein-like decoys. This simplified function correctly classified 408 native sequences (20 misclassifications, 95% correct rate), which outperforms several other statistical linear scoring function and optimized linear function. Our results further suggested that for the task of global sequence design of 428 selected proteins, the search space of protein shape and sequence can be effectively parametrized with just about 3,680 carefully chosen basis set of proteins and decoys, and we showed in addition that the overall landscape is not overly sensitive to the specific choice of this set. Our results can be generalized to construct other types of fitness landscape.

## Introduction

Protein design has been the focus of many experimental, theoretical, and computational studies [1]–[9]. Despite significant challenges, important progresses have been made, with profound implications in biotechnology and biomedicine [10]–[15].

Here we studied the problem of designing a protein sequence that is compatible with an *a priori* specified three-dimensional template protein fold. This problem was first formulated 30 years ago [16], [17]. Also known as the inverse protein folding problem, it addresses the fundamental problem of designing proteins to facilitate engineering of proteins with enhanced or novel biochemical functions.

A key component for designing a protein sequence is a fitness function: it can detect if a solution has been found, and can also guide the search of viable sequences. An ideal fitness function can characterize the properties of fitness landscape of many proteins simultaneously. Such a fitness function would be useful for designing novel proteins and novel functions, as well as for studying the global evolution of protein structure and protein functions.

The development of a fitness function for protein design is closely related to the development of a scoring function for protein structure predictions, protein folding, and protein-protein/ligand docking [18]–[23]. There are many different approaches in constructing the fitness function. Several studies employ a linear fitness function in the form of weighted linear sum of pairwise contacts, with sometimes additional solvation terms derived from exposed surface area [2], [3], [5]. Such functions can be obtained from statistical analysis of a database of protein structures [24], or from perceptron learning/linear programming [21], [25], [26], or by gradient descent [27], [28]. Another approach is to use a force field such as those used in molecular dynamics simulations [6], [29]–[31]. However these functions often do not provide global characterization of the overall fitness landscape for protein design. They also often have poor performance in blind test when challenged with the task of designing simultaneously many different proteins [32], or are so complex that they can not be used in high-throughput test. Inaccurate fitness functions can lead to low success rates in protein design [33].

A promising alternative approach is to use nonlinear function to capture the complex design of fitness landscape. In the study of [32], a nonlinear Gaussian kernel function was constructed by maximizing soft margins between native proteins and decoy non-proteins. This fitness function significantly outperforms linear functions in a blind test of identifying 201 native proteins from 3 million challenging protein-like decoys [32]. However, it is parametrized by about 350 native proteins and 4,700 non-protein decoys and its form is rather complex. It is computationally expensive to evaluate the fitness of a candidate sequence. Although obtaining a good answer at high computational cost is acceptable for some tasks, it is difficult to incorporate a complex function in a search algorithm. It is also difficult to characterize global landscape properties of protein sequence design using a complex function.

In this study, we demonstrated how to significantly improve nonlinear function for characterizing fitness landscape of protein design. Using a rectangular kernel with proteins and decoys chosen *a priori*, we obtained a nonlinear kernel function via a finite Newton method. The total number of native proteins and decoy conformations included in the function was reduced to about 3,680. In the blind test of sequence design to discriminate 428 native sequences from 11 million challenging protein-like decoy sequences, this fitness function misclassified only 20 native sequences (correct rate 95%), which far outperform statistical function [34] (87 misclassification, correct rate 57%) and linear optimal functions [26], [28] (44–58 misclassification, correct rate 78%–71%) both of which were tested on a smaller scale to discriminate 201 native sequence from 3 million challenging protein-like decoy sequences. It is also comparable to the results of 18 misclassification (correct rate 91%) using far more complex nonlinear fitness function with >5,000 terms [32].

This paper is organized as follows. We first describe our theory and methods for sequence design. We then discuss computational details. Results of a blind test are then presented. We conclude with discussion and remarks.

## Theory and Methods

We use a 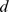-dimensional vector 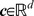 to represent both the sequence and structure of a protein [35]. One possible choice is the vector of the number count of non-bonded pairwise contacts of each of the 
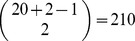
 contact types [24] between the 20 types of amino acid residues in a protein structure. Once the structural conformation of a protein 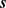 and its amino acid sequence 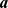 is given, the contact definition 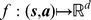 fully determines the contact vector 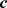.

### Inequality criterion

In protein design, the native amino acid sequence 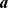 of a protein should have better fitness score on the native structure 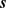 of this protein than any other competing sequences taken from proteins of different fold. This leads to the requirement that the native sequence 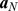 mounted on its native structure 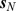 should have the best fitness score (lowest “energy”) compared to a set of decoys 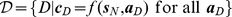 derived from mounting unrelated alternative sequences 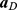 on the native protein structure 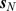: 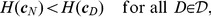
(1)where 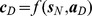 is the contact vector of a decoy sequence 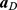 mounted on its native protein structure 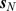, and 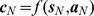 is the contact vector of a native sequence 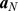 from the set of native training proteins 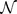 mounted on the native structure 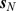. Here 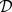 is a set of sequence decoys mounted on native protein structures. 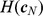 and 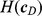 are the energy score for native sequence structure pair and for non-native sequence structure pair, respectively. Equivalently, the native sequence will have the highest probability to fit into its native structure, and other sequences will have lower probability. This is the same principle described in [3]–[5].

A commonly used form for fitness function 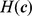 is the weighted linear sum of pairwise contacts [24], [26], [36]–[38]: 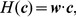
(2)here “

” represents inner product of two vectors. For such a linear function, the basic requirement for protein fitness is then: 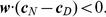
(3)


We can further require that the difference in fitness must be greater than a constant 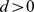: 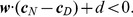
(4)


### Geometric views of inequality requirement

There is a natural geometric view of the inequality requirement. Each of the inequalities divides the space of 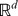 into two halves separated by a hyperplane. The hyperplane is defined by the normal vector 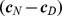 and its distance 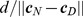 from the origin. The weight vector 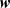 must be located in the half-space opposite to the direction of the normal vector 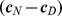. This half-space can be written as 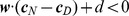. When there are many inequalities to be satisfied simultaneously, the intersection of the half-spaces forms a convex polyhedron [39]. If the weight vector is located in the interior of the polyhedron, all inequalities are satisfied. Fitness function with such weight vector 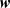 can discriminate a native protein from all decoys.

For each native protein 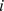, there is one convex polyhedron 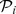 formed by the set of inequalities associated with its decoys. If the scoring function can discriminate simultaneously 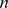 native contact vectors from a union of sets of decoys, the weight vector 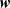 must be located in the interior of a smaller convex polyhedron 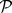 that is the intersection of the 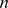 convex polyhedra: 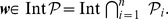


There is another geometric view of the inequality requirements. The relationship 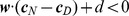 for all decoys and native protein sequences can be regarded as a requirement that all points 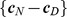 are located on one side of a hyperplane, which is defined by its normal vector 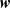 and its distance 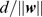 to the origin. We can show that such a hyperplane exists if and only if the origin is not contained within the convex hull of the set of points 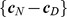 [32]. This second geometric view is dual to the first geometric view.

### Relation to support vector machines

There may exist multiple 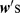 if 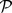 is not empty. We can use the formulation of a support vector machine to find a 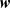. Let all vectors 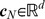 form a native training set and all vectors 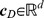 form a decoy training set. Each vector in the native training set is labeled as 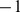 and each vector in the decoy training set is labeled as 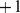. Then solving the following support vector machine problem will provide an optimal solution to inequalities (3): 
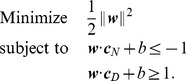
(5)


Note that a solution of the above problem satisfies the system of inequalities (3), since subtracting the second inequality from the first inequality in the constraint conditions of (5) will give us 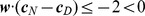.

### Nonlinear fitness function

However, it is possible that no weight vector 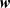 exists, *i*.e., the interior of the final convex polyhedron 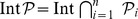 may be an empty set. First, for a specific native protein 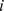, there may be severe restriction from some inequality constraints, which makes 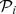 an empty set. Some decoys are very difficult to discriminate due to perhaps deficiency in protein representation. In these cases, it is impossible to adjust the weight vector so the native structure has a better fitness score than the decoy. Second, even if a weight vector 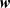 can be found for each native protein, *i*.e., 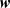 is contained in a nonempty polyhedron, it is still possible that the intersection of the interior of 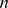 nonempty polyhedra is an empty set, *i*.e., no weight vector can be found that can make all native proteins simultaneously the fittest against decoys.

A fundamental reason for this failure is that the functional form of linear sum of pairwise interaction is too simplistic. To resolve this issue, we obtain nonlinear fitness function for sequence design using an alternative functional form [32]: 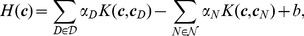
(6)where 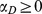 and 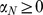 are coefficients to be determined. This functional form is reminiscent of the linear fitness function 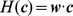, which can be written alternatively as an expansion around positive and negative contact vectors, as used in perceptron learning: 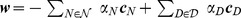. A convenient kernel function 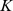 is: 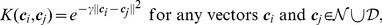
(7)where 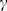 is a constant. The fitness function 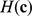 can be written compactly as: 
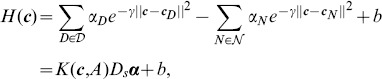
(8)where 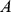 is the matrix of training data: 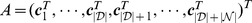, and the entry 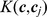 of 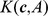 is 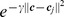. 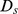 is the diagonal matrix with 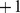 and 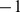 along its diagonal representing the membership class of each point 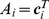. Here 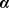 is the coefficient vector: 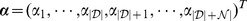.

Intuitively, the fitness landscape has smooth Gaussian hills of height 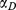 centered on location 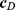 of decoy contact vector 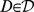, and has smooth Gaussian cones of depth 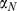 centered on the location 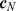 of native contact vector 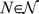. Ideally, the value of the fitness function will be 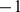 for contact vectors 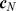 of native proteins, and will be 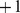 for contact vectors 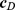 of decoys.

### Optimal nonlinear fitness function

To obtain such a nonlinear function, our goal is to find a set of parameters 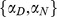 such that 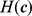 has fitness value close to 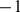 for native proteins, and has fitness values close to 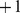 for decoys. There are many different choices of 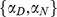. We use an optimality criterion developed in statistical learning theory [40]–[42]. First, we note that we have implicitly mapped each protein and decoy from 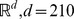 to another high dimensional space where the scalar product of a pair of mapped points can be efficiently calculated by the kernel function 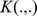. Second, we find the hyperplane of the largest margin distance separating proteins and decoys in the space transformed by the nonlinear kernel [40]–[43]. That is, we search for a hyperplane with equal and maximal distance to the closest native protein sequence and the closest decoys. Such a hyperplane has good performance in discrimination [40]. It can be found using support vector machine by obtaining the parameters 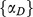 and 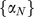 from solving the following primal form of quadratic programming problem:
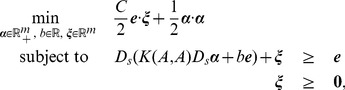
(9)where 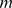 is the total number of training points: 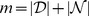, 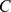 is a regularizing constant that limits the influence of each misclassified conformation [40]–[43], and the 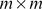 diagonal matrix of signs 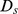 with 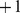 or 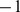 along its diagonal indicating the membership of each point 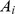 in the classes 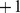 or 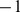; and 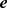 is an 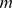-vector with 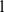 at each entry. The variable 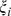 is a measurement of error for each input vector with respect to the solution: 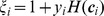, where 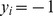 if 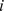 is a native protein, and 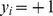 if 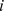 is a decoy.

### Rectangle kernel and reduced support vector machine (RSVM)

The use of nonlinear kernels on large datasets typically demands a prohibiting size of the computer memory in solving the potentially enormous unconstrained optimization problem. Moreover, the representation of the landscape surface using a large data set requires costly storage and computing time for the evaluation of a new unseen contact vector 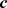. To overcome these difficulties, the reduced support vector machines (RSVM) developed by Lee and Mangasarian [44] use a very small random subset of the training set to build a rectangular kernel matrix, instead of the use of the conventional 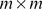 kernel matrix 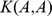 in equation (9). This model can achieve about 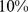 improvement on test accuracy over conventional support vector machine with random data sets of sizes between 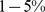 of the original data [44]. The small subset can be regarded as a basis set in our study. Suppose that the number of contact vectors in our basis set is 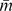, with 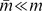. We denote 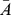 as an 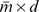 matrix, and each contact vector from the basis set is represented by a row vector of 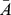. The resulting kernel matrix 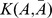 from 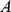 and 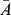 has size 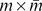. Each entry of this rectangular kernel matrix is calculated by 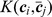, where 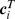 and 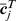 are rows from 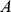 and 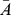 respectively. The RSVM is formulated as the following quadratic program: 
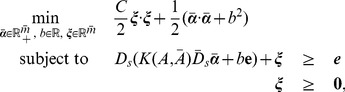
(10)where 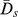 is the 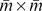 diagonal matrix with 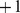 or 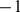 along its diagonal, indicating the membership of each point 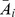 in the classes 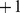 or 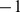; and 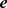 is an 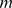-vector with 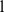 at each entry. As shown in [44], the zero level set surface of the fitness function is given by 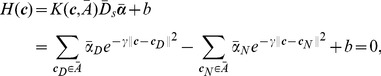
(11)where 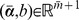 is the unique solution to (10). This surface discriminates native proteins against decoys. Besides the rectangular kernel matrix, the use of 2-norm for the error 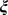 and an extra term 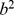 in the objective function of (10) distinguish this formulation from conventional support vector machine.

### Smooth Newton method

In order to solve equation (10) efficiently, an equivalent unconstrained nonlinear program based on the implicit Lagrangian formulation of (10) was proposed in [45], which can be solved using a fast Newton method. We modified the implicit Lagrangian formulation and obtain the unconstrained nonlinear program for the imbalance RSVM in equation (10). The Lagrangian dual of (10) is now [46]: 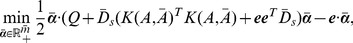
(12)where 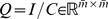, and 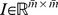 is unit matrix. Note that 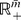 is the set of nonnegative 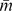-vectors. Following [45], an equivalent unconstrained piecewise quadratic minimization problem of the above positively constrained optimization can be derived as follows: 
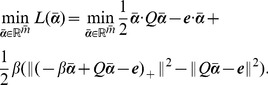
(13)


Here, 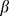 is a sufficiently large but bounded positive parameter to ensure that the matrix 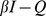 is positive definite, where 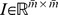 is a unit matrix, and the plus function 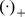 replaces negative components of a vector by zeros. This unconstrained piecewise quadratic problem can be solved by the Newton method in a finite number of steps [45]. The Newton method requires the information of the gradient vector 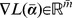 and the generalized Hessian 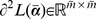 of 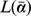 at each iteration. They can be calculated using the following formulae [45]: 
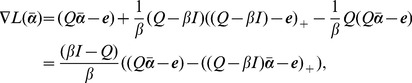
(14)and 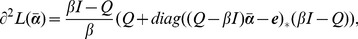
(15)where 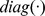 denotes a diagonal matrix and 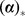 denotes the step function, *i*.e., 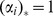 if 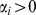; and 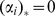 if 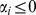.

The main step of the Newton method is to solve iteratively the system of linear equations 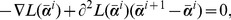
(16)for the unknown vector 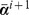 with given 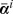.

We present below the algorithm, whose convergence was proved in [45]. We denote 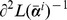 as the inverse of the Hessian 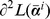.

Start with any 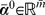. For 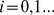:

- Stop if 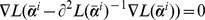.

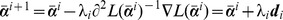
, where 
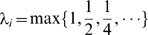
 is the Armijo step size [47] such that 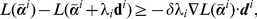
(17)for some 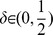, and 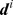 is the Newton direction 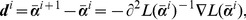
(18)obtained by solving (16).

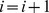
. Go to (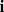).

## Computational Experiments

### Determination of count vector by alpha shape

Since protein molecules are formed by thousands of atoms, their shapes are complex. In this study, we use the count vector 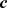 of pairwise contact interactions derived from the edge simplexes of the alpha shape of a protein structure, where only nearest neighbor atoms in physical contacts are identified. The advantages of this approach are elaborated in [48]. We refer to references [49], [50] for further theoretical and computational details.

### Relationship between number of contacts and length of protein

We found that there is a relationship between the number of total contacts of a protein and the length of the protein. A linear regression on the relationship between the number of total contacts and the length of the protein gives the following equation, 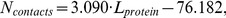
(19)where 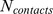 is the number of contacts for a protein, and 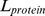 is the number of the protein residues. To eliminate the influence of the length of protein, we normalize the number of contacts for each type of pair-wise contact of a protein using equation (19).

### Generating sequence decoys by threading

**Figure 1:**
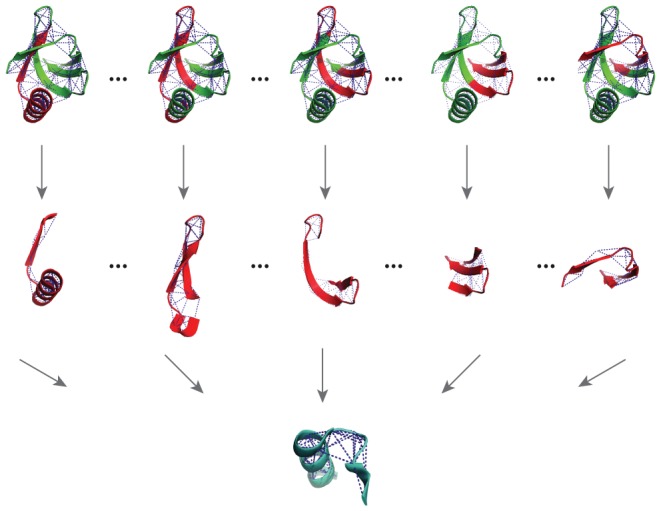
Decoy generation by gapless threading. Sequence decoys can be generated by threading the sequence of a larger protein to the structure of an unrelated smaller protein.

We followed Maiorov and Crippen [51] and used gapless threading to generate a large number of decoys for a simplified test of protein design. We threaded the sequence of a larger protein through the structure of a smaller protein, and obtained sequence decoys by mounting a fragment of the native sequence from the large protein to the full structure of the small protein. We therefore had a set of sequence decoys 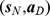 for each native protein 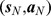 (Fig 1). Because all native contacts were retained, such sequence decoys are quite challenging. This is unlike folding decoys generated by gapless threading [32].

### Dataset

We used the list of 1,515 protein chains compiled from the PISCES server [52]. Protein chains in this data set have pairwise sequence identity 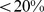, With its structural resolution by crystallography and has a resolution 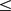 1.6 Å, and the R-factor 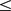 0.25. We removed incomplete proteins (i.e. those with missing residues), and proteins with uncertain residues (those denoted as ASX, GLX, XLE, and XAA). We further removed proteins with less than 46 and more than 500 amino acids. In addition, we removed protein chains with more than 30% extensive inter-chain contacts. The remaining set of 1,228 proteins are then randomly divided into two sets. One set includes 800 proteins and the other one includes 428 proteins. Using the sequence threading method, we generated 36,823,837 non-protein decoys, together with 800 native proteins as the training set, and 11,144,381 decoy non-proteins with 428 native proteins as the test set.

### Selection of matrix 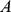 for iterative training

We used only a subset of the 36 million decoys and native structures so they could fit into the computer memory during training. These structures formed the data matrix 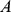, which was used to construct the kernel matrix 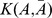. We used a heuristic iterative approach to construct matrices 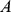 and 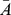 during each iteration.

Initially, we randomly selected 10 decoys from the set of decoys 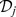 for each of the 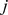-th native protein. We have then 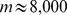 decoys for the 800 native proteins. We further chose only 1 decoy from the selected 10 decoys for each native protein 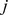. These 800 decoys were combined with the 800 native proteins to form the initial matrix 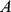. The contact vectors of a subset of 480 native proteins (60% of the original 800 proteins) and 320 decoys (40% of the 800 selected decoys) were then randomly chosen to form 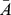. An initial fitness function 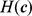 was then obtained using 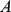 and 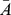. The fitness values of all 36 million decoys and the 800 native proteins were then evaluated using 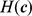. We further used two iterative strategies to improve upon the fitness function 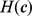.

[**Strategy 1**] In the 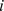-th iteration, we selected the subset of misclassified decoys from 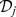 associated with the 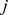-th native protein and sorted them by their fitness value in descending order, so the misclassified decoys with least violation, namely, negative but smallest absolute values in 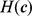, are on the top of the list. If there is less than 10 misclassified decoys, we add top decoys that were misclassified in the previous iteration for this native protein, if they exist, such that each native protein has 10 decoys.

A new version of the matrix 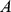 was then constructed using these 8,000 decoys and the corresponding 800 native proteins. To obtain the updated 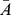, from these 8,800 contact vectors, we randomly selected 480 native proteins (60%) and 3,200 unpaired decoy non-proteins (40%) to form 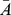.

The iterative training process was then repeated until there is no improvement in the classification of the 36 million decoys and the 800 native proteins from the training set. Typically, the number of iterations was about 10. In subsequent studies, we experimented with different percentage of selected decoys, ranging from 10% to 100% to examine the effect of the size of 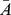 on the effectiveness of the fitness function 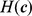.

[**Strategy 2**] In the 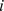-th iteration, we selected the top 10 correctly classified decoys sorted by their fitness value in ascending order for each native protein, namely, those correctly classified decoy with positive but smallest absolute values are selected. These contact vectors of 8,000 selected decoys are combined with the 800 native proteins to form the new data matrix 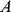.

To construct 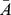, we first selected the most challenging native proteins by taking the top 80 correctly classified native proteins (10%) sorted by their fitness value in descending order, namely, those that are negative but with smallest absolute values in 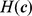. We then randomly took 400 native proteins (50%) from the rest of the native protein set, so altogether we have 480 native proteins (60%). Similarly, we selected the top one decoy that is most challenging from the 10 chosen decoys in 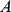 for each native protein, namely, the top decoy that is correctly classified with positive but smallest value of 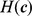. We then randomly selected 3 decoys for each native protein from the remaining decoys in 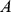 to obtain 3,200 decoy non-proteins (40%). The matrix 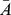 is then constructed from the selected 480 native proteins and 3,200 decoy non-proteins. The iterative training process was repeated until there was no improvement in classification of the 36 million decoys and 800 native proteins in the training set. Typically, the number of iteration was about 5.

In the subsequent studies, we evaluated our method with different choices of challenging native proteins. The selection ranges from the top 10% to 60% most challenging native proteins. The choice of the challenging decoys was also varied, where we experimented with choosing the top one to the top four most challenging decoys for each native protein, while the number randomly selected decoys varies from three to zero.

### Learning parameters

There are two important parameters: the constant 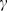 in the kernel function 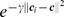, and the cost factors 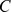, which is used during training so errors on positive examples were adjusted to outweigh errors on negative examples. Our experimentation showed that 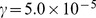 and 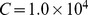 are reasonable choices.

### Running time

The algorithm was implemented in the C language. It called Lapack [53] and used LU decomposition to solve the system of linear equations. It also called an SVD routine to determine the 2-norm of a matrix for calculating 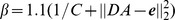. Once matrices 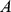 and 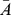 were specified, the fitness function 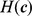 can be derived in about 2 hours and 10 minutes on a 2 Dual Core AMD Opteron(tm) Processors of 1,800 MHz with 4 Gb memory for an 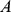 of size 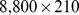 and an 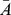 of size 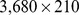. The evaluation of the fitness of 14 million decoys took 2 hours and 10 minutes using 144 CPUs of a Linux cluster (2 Dual Core AMD Opteron(tm) Processors of 1.8 GHz with 2 Gb memory for each node). Because of the large size of the data set, the bottleneck in computation is disk IO.

## Results

### Performance in discrimination

We used the set of 428 natives proteins and 11,144,381 decoys for testing the designed fitness function. We took the sequence 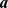 for a protein such that 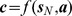 has the best fitness value as the predicted sequence. If it is not the native sequence 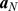, then the design failed and the fitness function did not work for this protein.

Sequence decoys obtained by gapless threading were quite challenging, since all native contacts of the protein structures were maintained, and decoy sequences were from real proteins. In a previous study, we showed that no linear fitness function can be found that would succeed in the challenging task of identifying all 440 native sequences in the training set [32]. Because we are unaware of any other development of design fitness functions amenable for high-throughput tests, and frequently no distinctions were made between protein folding potential and protein design fitness function, we compared our fitness function with several well-established scoring functions developed for protein folding.

We also use the 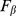 score to evaluate the performance of predictions. 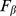 is defined as:
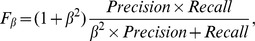
where 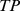 is the number of true positives, 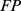 the number of false positives, 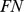 the number of false negatives, 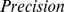 is calculated as 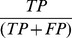, and 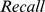 is calculated as 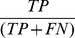. When 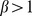, recall is emphasized over precision. When 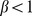, precision is emphasized over recall. Because of the imbalanced nature of the data set with much more decoys than native proteins, we assign more weight on the small set of native proteins, with 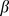 set to 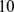. The 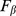 scores are than calculated accordingly.

Here we succeeded in obtaining a simplified nonlinear fitness function for protein design that are capable of discriminating 796 of the 800 native sequences (Table 1). It also succeeded in correctly identifying 95% (408 out of 428) of the native sequences in the independent test set. Results for other methods were taken from literature obtained using much smaller and less challenging data set. Overall, the performance of our method is better than results obtained using the optimal linear scoring function taken as reported in [26] and in [28], which succeeded in identifying 78% (157 out of 201) and 71% (143 out of 201) of the test set, respectively. Our results are also better than the Miyazawa-Jernigan statistical potential [34] (success rate 58%, 113 out of 201). This performance is also comparable with a more complex nonlinear fitness function, with 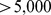 terms reported in [32], which succeeded with a correct rate of 91% (183 out of 201).

**Table 1:** The number of misclassification compared with other methods.

Method	Training set	Training set	Test set	Test set
	800 / 36 M	440 / 14 M	428 / 11 M	201 / 3 M
Nonlinear function	4 / 988	NA	20 / 218	NA
Tobi *et. al.*	NA	192 / 39,583	NA	44 / 53,137
Bastolla *et. al.*	NA	134 / 47,750	NA	58 / 29,309
Miyazawa & Jernigan	NA	173 / 229,549	NA	87 / 80,716

The number of misclassification using simplified nonlinear fitness function, optimal linear scoring function taken as reported in [26], [28], and Miyazawa-Jernigan statistical potential [34] for both native proteins and decoys (separated by “/”) in the test set and the training set. The simplified nonlinear function is formed using a basis set of 3,680 (480 native+3,200 decoy) contact vectors derived using Strategy 2.

### Effect of the size of the basis set 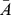 using Strategy 1

The matrix 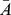 contains both proteins and decoys from 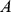 and its size is important in discrimination of native proteins from decoys. In our fitness function, Gaussian kernels centered around these selected contact vectors were used as basis set to interpolate the global landscape of protein design.

We examined the effects of different sizes of 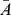 using Strategy 1. For a data matrix 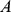 consisting of 800 native proteins and 8,000 sequence decoys derived following the procedure described earlier, we tested different choice of 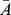 on the performance of discrimination. With the data matrix 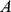, we fixed the selection of the 480 native proteins (60%), and experimented with random selection of different number of decoys, ranging from 800 (10%) to 8,000 (100%) to form different 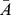
s.

The results of classifying both the training set of 800 native proteins with 36 million decoys and the test set of 428 native proteins with 11 million decoys are shown in Table 2. When 60% (480) native proteins and 100% (8,000) decoys are included, there are only 5 native proteins misclassified in the training set and 24 native proteins in the test set.

**Table 2:** Effects of the size of basis set 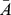 on performance of discrimination using Strategy 1.

		Training set		Test set	
Select decoys rate	Iteration	Native / Decoy	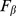	Native / Decoy	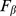
		800 / 36 M		428 / 11 M	
0%	4	21 / 1,374	0.958	26 / 387	0.931
2%	5	19 / 1,029	0.964	27 / 219	0.933
5%	5	17 / 1,303	0.963	21 / 317	0.944
8%	5	13 / 1,246	0.969	23 / 274	0.941
10%	5	14 / 922	0.972	24 / 216	0.940
20%	6	16 / 902	0.969	28 / 250	0.930
30%	6	10 / 1,037	0.975	29 / 304	0.926
40%	10	16 / 812	0.970	27 / 199	0.933
50%	10	13 / 1,112	0.971	25 / 269	0.936
60%	12	15 / 802	0.972	27 / 237	0.932
70%	9	13 / 947	0.973	24 / 256	0.939
80%	8	11 / 1,078	0.973	28 / 278	0.929
90%	9	12 / 690	0.977	27 / 170	0.934
100%	5	5 / 2,681	0.962	24 / 609	0.931

The number of misclassifications of both native proteins and decoys (separated by “/”) with select native proteins rate 60% in both training set and test set are listed. Misclassifications as well as the 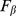 scores in two tests using different number of native proteins and decoys are listed (see text for details).

### Effect of the size of the pre-selection of dataset using Strategy 2

We examined the effects of different choices in constructing matrix 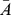 using Strategy 2. We varied our selection of the most challenging native proteins from the top 10% to 60%, and varied selection of the most challenging decoys from the top one to the top four decoys for each native protein, as describe earlier. Results are shown in Table 3. We found that the performance of the discrimination of both the training set and test set have little changes when either native proteins selection rate is changed from 10% to 60%, or decoys selection rate is changed from the top 1 to the top 4. Overall, these results suggest that for the blind test developed here, a fitness function with good discrimination can be achieved with about 480 native proteins and 3,200 decoys, along with 400 pre-selected native proteins and 800 pre-selected top-1 decoys. Our final fitness function used in Table 1 is constructed using a basis set of 3,680 contact vectors. We also observed that the average number of iterations is about 5 using Strategy 2, which is much faster than Strategy 1.

**Table 3:** Effect of the size of the pre-selection of dataset using Strategy 2.

			Training Set		Test set	
Pre-select native proteins top	Pre-select decoys top	Iteration	Native / Decoy	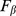	Native / Decoy	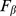
			800 / 36 M		428 / 11 M	
0%	1	6	8 / 1,010	0.978	25 / 212	0.938
2%	1	5	5 / 1,079	0.981	24 / 266	0.939
5%	1	5	5 / 1,038	0.981	24 / 247	0.939
8%	1	5	5 / 1,093	0.981	24 / 249	0.939
10%	1	5	5 / 997	0.982	24 / 242	0.939
20%	1	6	9 / 625	0.981	26 / 174	0.936
30%	1	6	9 / 689	0.980	24 / 211	0.940
40%	1	6	8 / 869	0.980	25 / 218	0.937
50%	1	5	4 / 988	0.983	20 / 218	0.949
60%	1	5	6 / 1,039	0.980	24 / 280	0.938
10%	1	5	5 / 997	0.982	24 / 242	0.939
10%	2	5	6 / 1,270	0.977	22 / 372	0.941
10%	3	7	9 / 934	0.978	22 / 247	0.944
10%	4	5	5 / 1,071	0.981	24 / 210	0.944

Test results using Strategy 2 with different sizes of the pre-selected native proteins, which range from 0% to 60% while the pre-selected decoys are fixed as the top 1 level, and with different pre-selected decoys, which ranges from the top 1 s to the top 4 s while the pre-selected native proteins are fixed at 10%. Misclassifications as well as the 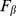 scores in two tests using different number of native proteins and decoys are listed (see text for details).

We found that using Strategy 2 (Table 3) leads to overall better performance compare to using Strategy 1 (Table 2). Specifically, the fitness function formed by pre-selecting the top 1 decoys and top 50% native proteins using Strategy 2 works well to discriminating native proteins from decoys.

Furthermore, our method is robust. The overall performance using either Strategy 1 or Strategy 2 is stable when decoy selection rate changes from 5% to 90%. Using the 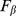 score as the criterion, we found that using Strategy 2 gives significantly more accurate result than using Strategy 1.

### Discrimination against a different decoy set

**Figure 2:**
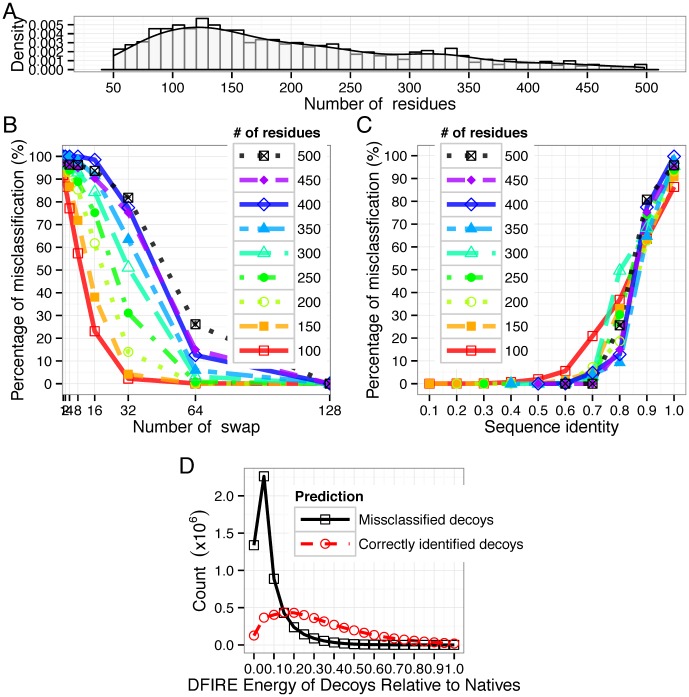
Discriminating a different decoy set using the nonlinear fitness function. Sequence decoys in this set are generated by swapping residues at different positions. (A). The length distribution of the 1,227 native proteins in the set; (B). The relationship between the number of swaps 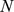 and the percentage of misclassified decoys grouped by protein length binned with a width of 50 residues shown in different curves. (C). The relationship between the sequence identity binned with width 0.1 and the percentage of misclassification grouped by protein length shown in different curves. The fitness function was derived using strategy 2, with top 50% pre-selected native proteins, and top 1 pre-selected decoys. (D). Misclassified sequence decoys have overall lower DFIRE energy values than correctly classified sequence decoys and therefore are more native-like. The 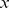-axis is the net DFIRE energy difference of decoys to native proteins, and the 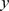-axis is the number count of decoys at different net DFIRE energy differences. The solid black line represents decoys misclassified by our fitness function and the dashed red line represents decoys correctly classified by our fitness function.

To further assess our fitness function, we examine how well decoys generated by a different approach can be discriminated using our nonlinear fitness function. We selected 799 training proteins and 428 test proteins for this test. Figure 2A shows the length distribution of these 1,227 proteins. To generate these new decoys, we fixed the composition of each of these proteins and permute its sequence by carrying out 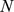 swaps between random residues, with 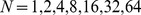, and 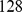. The resulting decoys all have the same amino acid composition as the original native proteins, but have progressively more point mutations. We generate 1,000 random sequence decoys at each swap 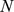 for each protein. We call this Decoy Set 2.

Our results show that as expected, the number of misclassified decoys decreases rapidly as the number of swaps increases. When 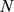 increase from 1 to 32, The percentage of misclassified decoys for protein of length 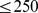 is about 30% or less. Less than 30% of the decoys of all lengths are misclassified when 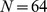, with the rate of misclassification much smaller than 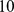
% among those with length 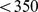 (Fig 2B). Only 62 decoys are misclassified among 1,227,000 decoys when 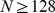 (Fig 2B).

It is informative to examine the number of misclassified decoys and the sequence identity of the decoys with their corresponding native proteins at different protein lengths. Figure 2C shows that the percentage of misclassified decoys decreases rapidly with the sequence identity to the native proteins. When decoys have a sequence identity of 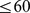
% with the native protein, 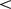 10% of the decoys are misclassified, and all decoys can be discriminated against at 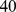
% identity for proteins of length 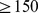. For proteins of length 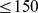, most decoys with 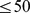
% sequence identity can be corrected discriminated against. These observations are consistent with current understanding of protein structures, where most proteins with 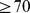
% sequence identity belong to the same family [54], and these with 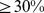 sequence identity have similar structure [55].

To examine whether misclassified decoy sequences are actually more native-like and therefore more likely to potentially adopt the correct structures than those correctly classified as non-natives, we selected 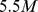 misclassified decoys and 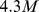 correctly classified decoys from all decoys in Decoy Set 2, and examined their energy values. We use the DFIRE energy function that was independently developed in [56], [57]. These decoys all have values of net DFIRE energy difference of decoys to native proteins within the interval of [0.0, 1.0] kcal/mol. Our results (Fig 2D) show that overall, misclassified decoys have much lower average DFIRE energy values, indicating that they are potentially more native-like than those correctly classified as decoys.

## Discussion

In this study, we have developed a simplified nonlinear kernel function for fitness landscape of protein design using a rectangular kernel and a fast Newton method. The results in a blind test are encouraging. They suggest that for a simplified task of designing simultaneously 428 proteins from a set of 11 million decoys, the search space of protein shape and sequence can be effectively parametrized with just about 3,680 basis set of contact vectors. It is likely that the choice of matrix 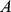 is important. We showed that once 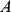 is carefully chosen, the overall design landscape is not overly sensitive to the specific choice of the basis set contact vectors for 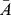.

The native protein list in both training and test sets come from the PISCES server, which has the lowest pair-wise identity (20%), finer resolution cutoff (1.6 Å), and lower R-factor cutoff (0.25). This native dataset is better than previous study [32] dataset derived from the WHATIF database, which has looser constraints: pair-wise sequence identity 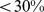, resolution cutoff <2.1 Å, and R-factor cutoff 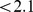. We compared our results with classic studies of Tobi *et. al.* [26], Bastolla *et. al.* [28] and Miyazawa and Jernigan [34]. Although the training set and test set are different, we observed that our simplified nonlinear function detected 95% (208) native proteins from 11 million decoys and only misclassified 218 decoys as native proteins, which outperformed Tobi *et. al.* [26] (78% correct rate for native proteins, 53,137 misclassification for decoys), Bastolla *et al.* [10] (71% correct rate for native proteins, 29,309 misclassification for decoys), and Miyazawa and Jernigan [34] methods (57% correct rate for native proteins, 80,716 misclassification for decoys) on much smaller blind test set of 201 native proteins and 3 million decoys.

As protein length is linearly correlated with the total number of contacts, we found that length corrections is important for improving fitness function. For example, the rate of misclassification is 7.2% in an earlier study without length correction (14 out 494 natives) [58], while this rate is now improved to 4.7% in the current study with length correction (20 out of 428 misclassified).

We developed two strategies to search for improving fitness landscape. Strategy 1 mostly uses misclassified decoys in the next iteration of construction of matrix 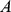. On average, 10 iterations is necessary to arrive at a good fitness function, which has excellent performance of only 5 misclassification for the training data set. The misclassification rate in the test set is comparable to other fitness function [26], [28], [34]. Strategy 2 selected the most challenging decoys by the fitness value landscape in the matrix 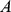 for the next iteration. We pre-selected certain percentage of the number of native proteins and certain number of decoys before generating the basis set matrix 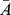. Overall, Strategy 2 performs better than Strategy 1, not only in reducing both native proteins and decoys misclassifications in the blind test set, but also can speed up the search process in deriving the final fitness function with the number of iteration reduced from 10 to 5 iterations. With Strategy 2, the updated fitness landscape is only adjusted by challenging decoys, it can identify the most challenging decoys and native proteins, leading to improved the fitness landscape in the next iteration.

Our final fitness landscape can correctly classify most of the native proteins, except 4 proteins (1ft5 chain A, 1gk9 chain A, 2p0s chain A, 2qud chain A) in the training set and 20 proteins in the test set (Table. 4). Among misclassified proteins, 4 of which have 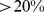 contacts due to inter chain interactions. In addition, 14 misclassified proteins contain metal ions and organic compounds. We note that the interactions between these organic compounds, metal ions and rest of the protein are not reflected in the protein description. It is likely that substantial unaccounted interactions with other protein chains, DNA, or co-factors contributed to the misclassifications. The conformations of these proteins may be different upon removal of these contacts. Altogether, 21 of the 24 misclassified proteins have explanations, and the fitness function truly failed only for 3 proteins.

**Table 4:** 20 native proteins in the test set are misclassified using Strategy 2.

Molecular name		Classification	Ligand(s)	PDBID	Chain	Fitness value
Catalase	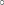	Oxidoreductase	1 HEM and 3 SO 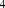	1gwe	A	0.1085
Streptavidin	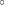	Biotin binding	1 BTN and 2 GOL	2f01	A	0.1407
Acutohaemonlysin	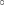	Toxin	2 IPA	1mc2	A	0.1728
Endonuclease I	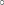	Hydrolase	1 Mg and 2 Cl	2pu3	A	0.1900
cytochrome c, putative	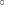	Electron transport	2 SO 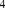 , 1 Na and 2 HEM	2czs	A	0.2664
Cytochrome F	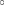	Electron transport	1 HEME C	1e2w	A	0.6023
Bowman-Birk type trypsin inhibitor	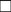	Hydrolase inhibitor	None	2fj8	A	0.8463
Uncharacterized protein with erredoxin-like fold	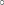	Structural genomics Unknown function	1 Unkown ligand	3e8o	A	1.1592
General secretion pathway protein G	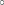	Protein transport	1 Zn	1t92	A	1.3175
ARF GTPase-activating protein git1	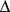	Signaling protein	None	2w6a	A	1.6581
Cystatin B	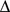	Protein binding	None	2oct	A	1.8043
SNAP-25A	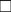	Transport protein	None	1n7s	D	1.9074
Lin2189 protein	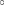	Structural genomics Unknown function	2 GOL	3b49	A	2.0142
Fibritin	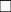	Chaperone	None	2ibl	A	2.1211
Oxalate oxidase 1	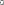	Oxidoreductase	1 Mn, 1 GLV	2et1	A	2.9975
Alpha-2-macroglobulin receptor-associated protein	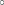	Lipid transport endocytosis chaperone	2 Ca, 1 Na and 3 MPD	2fcw	B	3.5660
Recombination endonuclease VII	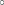	Plasma protein	1 Zn and 7 SO 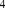	1e7l	A	3.7397
Hypothetical protein	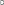	Isomerase	1 BEZ	1gyx	A	4.2697
YDCE	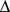					
Syntaxin 1a	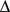	Transport protein	None	1n7s	B	5.0204
Bacteriophage t4 short tail fibre	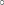	Structural protein	1 CIT, 2 SO 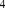 and 1 Zn	1ocy	A	8.0264

The number of ligands bound to the protein are listed. The molecules are sorted by the fitness value. 14 of them (marked by “
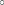
”) have ligand(s) bound to the protein. 4 of them (marked by “
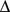
”) have 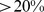 contacts due to inter chain interactions. The fitness function definitively failed for only 3 proteins (marked by “
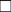
”). For the remaining 17 proteins, the contacts between organic compounds and metal ions with the protein and inter chain interactions may provide additional stability beyond the intra-residue interactions captured in the descriptors.

In protein folding studies, it is well known that contact maps of decoys formed by gapless threading have considerable higher energy than the native contact map, and these protein folding decoys are not as challenging as decoys generated by other methods such as Monte Carlo simulation. Results showed in Figure 2 demonstrated that these sequence decoys are challenging, and our nonlinear fitness function works well.

The representation of protein structures will likely have important effects on the success of protein design. The approach of the reduced nonlinear function is general and applicable when alternative representations of protein structures are used, *e.g.*, adding solvation terms, including higher-order interactions.

## Conclusions

We showed that a simplified nonlinear fitness function for protein design can be can be obtained using a simplified nonlinear kernel function via a finite Newton method. We used a rectangular kernel with a basis set of native proteins and decoys chosen *a priori*.

We succeeded in predicting 408 out of the 428 (95%) native proteins and misclassified only 218 out of 11 million decoys in a large blind test set. Although the test sets used is different, as other method were based on relatively small (201 native proteins and 3 million decoys) blind test set. Our result outperforms statical linear scoring function ( 87 out of the 201 misclassifications, 57% correct rate) and optimized linear function (between 44 and 58 misclassifications out of the 201, 78% and 71% correct rate). The performance is also comparable with results obtained from a far more complex nonlinear fitness function with 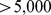 terms (18 misclassifications, 91% correct rate). Our results further suggest that for the task of global sequence design of 428 selected proteins, the search space of protein shape and sequence can be effectively parametrized with just about 3,680 carefully chosen basis set of native proteins and non-native protein decoys.

The rectangle kernel matrix with a finite Newton method works well in constructing fitness landscape. In addition, we showed that the overall landscape is not overly sensitive to the specific choice of the dataset.

Overall, our strategy of reduced kernel can be generalized to constructing other types of fitness function.
